# Threshold Limit Graphical Approach to Understanding Outcome Predictive Metrics: Data from the Osteoarthritis Initiative

**DOI:** 10.7759/cureus.1447

**Published:** 2017-07-08

**Authors:** John A Hipp, Elaine F Chan

**Affiliations:** 1 Research, Medical Metrics; 2 Technical Services, Medical Metrics

**Keywords:** threshold limit graph, associations between variables, joint space narrowing, symptoms

## Abstract

Scatter plots, bar charts, linear regressions, analysis of variance, and other graphics and tests are frequently used to document associations between an independent variable and an outcome. However, these methods are also frequently limited when understanding how to use an independent variable in subsequent research or patient management. A novel graphical approach to visualizing data—the threshold limit graph—was therefore developed.

Publically available data from the Osteoarthritis Initiative was used to illustrate the graphical approach to understanding the association between the change in joint space width (ΔJSW, independent variable) over four years, and knee symptoms at four years (using the Knee Injury and Osteoarthritis Outcome Score [KOOS], dependent variable).

Using data for 4,202 knees, the traditional scatter plot and linear regression approach showed a significant but weak linear relationship between the symptom subscore of the KOOS and ΔJSW. However, the threshold level of ΔJSW that affects symptoms was not clear from the data. The same dataset was then plotted using the threshold limit graphical approach, which revealed a non-linear relationship between the variables. In contrast to the scatter plot, plotting the average KOOS symptom subscore for subgroups of the data, with each subgroup defined using sequentially increasing or decreasing ΔJSW thresholds revealed that symptoms got worse with joint space loss, but only when there was a significant amount of ΔJSW. A threshold limit analysis was repeated using small, randomly selected subsets of the data (N = ~100) to demonstrate the utility of the technique for identifying trends in smaller datasets.

The threshold limit graph is a simple, graphical approach that may prove helpful in understanding how an independent variable might be used to predict outcomes. This approach provides an additional option for visualizing and quantifying associations between variables.

## Introduction

Regression equations, scatter plots, bar graphs, and associated statistics are frequently used to illustrate that a variable has a significant effect on clinical outcomes. However, based on these tools, it is often difficult to understand how an apparently predictive variable might be used to manage an individual patient. This technical report describes an approach to graphically displaying data that can help users better understand the effect of a variable on clinical outcomes. The approach is based on identifying any significant changes in outcomes that occur, as subgroups of the subjects are analyzed based on a threshold level of the independent variable. To the authors’ knowledge, there is no previously published literature using this methodology.

Publically available data from the Osteoarthritis Initiative (OAI) were analyzed for this report. The OAI is a nationwide research program sponsored by the National Institutes of Health (NIH) to study the treatment and prevention of knee osteoarthritis. The purpose of the analysis was to determine the effect of the change in joint space width (ΔJSW) over four years on the clinical outcome at four years, as determined by the Knee Injury and Osteoarthritis Outcome Score symptom subscore (KOOS) [1-2].

## Technical report

The data used in the preparation of this report was obtained from the OAI database after completing the required data use agreement. Data is available for public access at http://www.oai.ucsf.edu/. Specific datasets used were from JointSx06SAS.zip 26-Oct-2011, kXRQJSWDuryea00SAS.zip 11-Mar-2016, and kXRQJSWDuryea06SAS.zip 11-Mar-2016. These datasets include the reference, progression, and incident cohorts from the OAI study [3]. Data for a total of 4,202 knees from 2,506 subjects were analyzed. This data represents every subject from the OAI study that had available radiographic knee joint space width (JSW) measurements at baseline and four years, and KOOS symptom subscores at four years. JSW was measured at the location of minimum joint space [4-5], and ΔJSW was calculated as the difference in JSW between baseline and four years. A negative ΔJSW value represents joint space loss, and a positive value represents joint space gain over time. KOOS symptom subscores were reported as a normalized outcome in the OAI data such that a score of 100 indicated no symptoms and a score of 0 indicated extreme symptoms. All variables were reported separately for left and right knees.

The data was analyzed in Stata (version 14, Stata Corp, College Station, TX) using a custom script to calculate the average KOOS symptom subscore for subgroups defined by a threshold level of ΔJSW. For example, the average KOOS symptom subscore was calculated including all subjects with a ΔJSW < -1 mm. Average KOOS symptom subscores were then plotted as a function of the threshold limit used to define the subgroup (threshold limit graph). Those data were used to visualize the effect of ΔJSW on KOOS symptom subscore graphically. Bootstrap sampling was conducted in Stata to estimate the overall variability in the KOOS symptom subscore data for all 4202 knees. The 95% confidence interval from the bootstrap function was used to define the range of KOOS symptom subscores that can be explained by the overall variability in the data. Thus, a subgroup that has an average KOOS symptom subscore outside of the 95% confidence interval is considered to differ significantly from the overall population.

**Figure 1:**
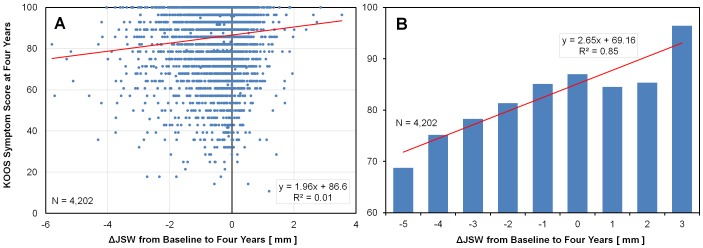
Scatter Plot & Bar Graph Display Traditional techniques for analyzing the relationship between average KOOS symptom subscale at four years versus ΔJSW from baseline to four years, based on the entire dataset representing 4,204 knees. (A) Scatter plot with a linear regression fit shows a significant but weak correlation between the KOOS symptom subscore and ΔJSW. (B) The same data plotted as a bar graph based on binning of ΔJSW values (excludes bars with fewer than two data points). Linear regression shows a strong correlation between KOOS symptom subscore and ΔJSW. Abbreviations: KOOS, Knee Osteoarthritis Outcome Score; ΔJSW, change in joint space width.

Using a traditional scatter plot approach, including all available data, no clear trend is evident between ΔJSW and KOOS symptom subscores (Figure 1A). From the scatter plot, it would appear that factors other than the ΔJSW account for variability in the KOOS symptom subscore. Nevertheless, a linear regression analysis indicates a significant (P < 0.0001) but weak (R^2^ = 0.01) relationship between clinical outcomes and ΔJSW. With a binned bar graph plot of the same data, the trend in lower KOOS symptom subscores with greater joint space loss becomes apparent (Figure 1B). However, it is unclear whether this trend is linear across the entire range of ΔJSW in the OAI data. This could be important in application of personalized medicine toward predicting prognosis based on a specific ΔJSW for a particular patient.

**Figure 2:**
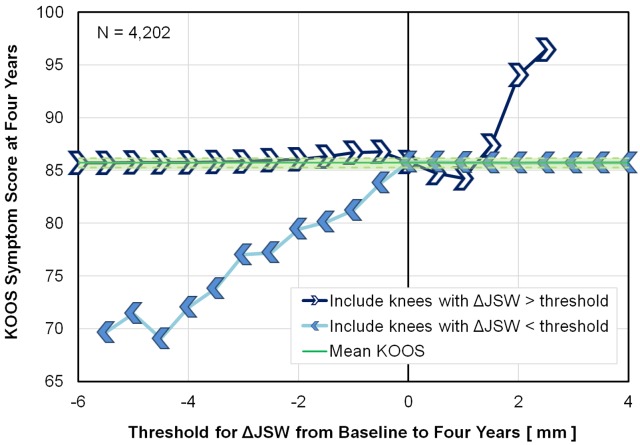
Threshold Limit Graph The threshold limit graph technique for analyzing the average KOOS symptom subscale at four years versus ΔJSW from baseline to four years, based on the entire dataset representing 4,204 knees. Each Chevron marker represents a subgroup of the data where ΔJSW was *greater than* the current ΔJSW threshold (dark blue line with open markers pointing right), or *less than* the current ΔJSW threshold (light blue line with filled markers pointing left). The green solid line represents the mean KOOS symptom subscore of the entire population, and the shaded green region represents the 95% confidence interval around the mean. Markers outside of the shaded green region are significantly different from the average of the entire population. The two curves provide complimentary data to understand the ΔJSW threshold at which patient outcomes are expected to be better or worse. Abbreviations: KOOS, Knee Osteoarthritis Outcome Score; ΔJSW, change in joint space width.

Figure 2 displays an alternative way to visualize the same data using the novel threshold limit graph. With this plotting technique, two complimentary series of data points depict clear trends in KOOS symptom subscores when ΔJSW goes above or below certain threshold limits. In the light blue series, with filled marker arrows pointing left (<), each marker represents the average KOOS symptom subscore of a subgroup that only includes those knees with ΔJSW *less than* the marker ΔJSW value (or the threshold limit). For example, the marker at -2 mm ΔJSW represents a subgroup of the population where only knees with ΔJSW < -2 mm were *included*, and ΔJSW ≥ -2 mm were excluded. Moving from right to left on the curve, the light blue series incrementally excludes greater amounts of data. In contrast, the dark blue series, with open marker arrows pointing right (>), only includes knees where ΔJSW was *greater than* the threshold limit at each marker. Thus, data is incrementally excluded moving from left to right on the curve. The horizontal green line shows the average KOOS symptom subscore at four years for the entire population, with the shaded green region depicting the 95% confident interval around the mean.

In the threshold limit graph, when the markers on either the light or dark blue series move above or below the 95% confidence interval for the entire population, the subgroup represented by the marker is significantly different from the entire population. Focusing on the light blue series, when only those subjects with ΔJSW < -0.5 mm are included, the KOOS symptom subscore is significantly below the average for the entire population. Using data for the markers on the light blue line that include only subjects with ΔJSW < -0.5 mm, a linear regression line supports that the KOOS symptom subscore decreases by three for every 1 mm loss in joint space (P < 0.0001, R^2^ = 0.95; data not shown). This graphical approach suggests there is an association between ΔJSW over four years and patient outcomes at four years, but *only *for patients that lost significant joint space. Interestingly, the dark blue series shows a trend of improved outcome score for patients with an *increase *in joint space over a 1.5-mm threshold; this association was not investigated further.

**Figure 3:**
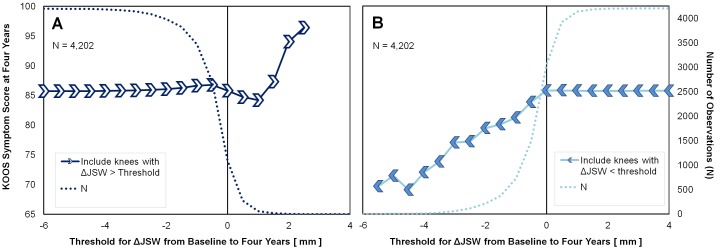
Sample Size Considerations The same graphs from Figure 2 with the addition of the number of observations used to calculate the subgroups at each marker. Each marker represents a subgroup of the data where ΔJSW was greater than the current ΔJSW threshold (A), or less than the current ΔJSW threshold (B). Abbreviations: KOOS, Knee Osteoarthritis Outcome Score; ΔJSW, change in joint space width.

It is important to appreciate that as an increasingly larger proportion of data is excluded, the sample size can get very small. Figure 3 contains the same data as Figure 2, with the addition of data showing how the sample size is affected by excluding data. It can be seen from Figure 3 that a relatively small proportion of the subjects lost > 2 mm or gained > 1 mm JSW. These graphs help to illustrate that ΔJSW may be predictive of outcomes, but only for those subjects with substantial changes in joint space. This is an observation that cannot be readily appreciated from a scatter plot or simple linear regression analysis of data for the entire population.

**Figure 4:**
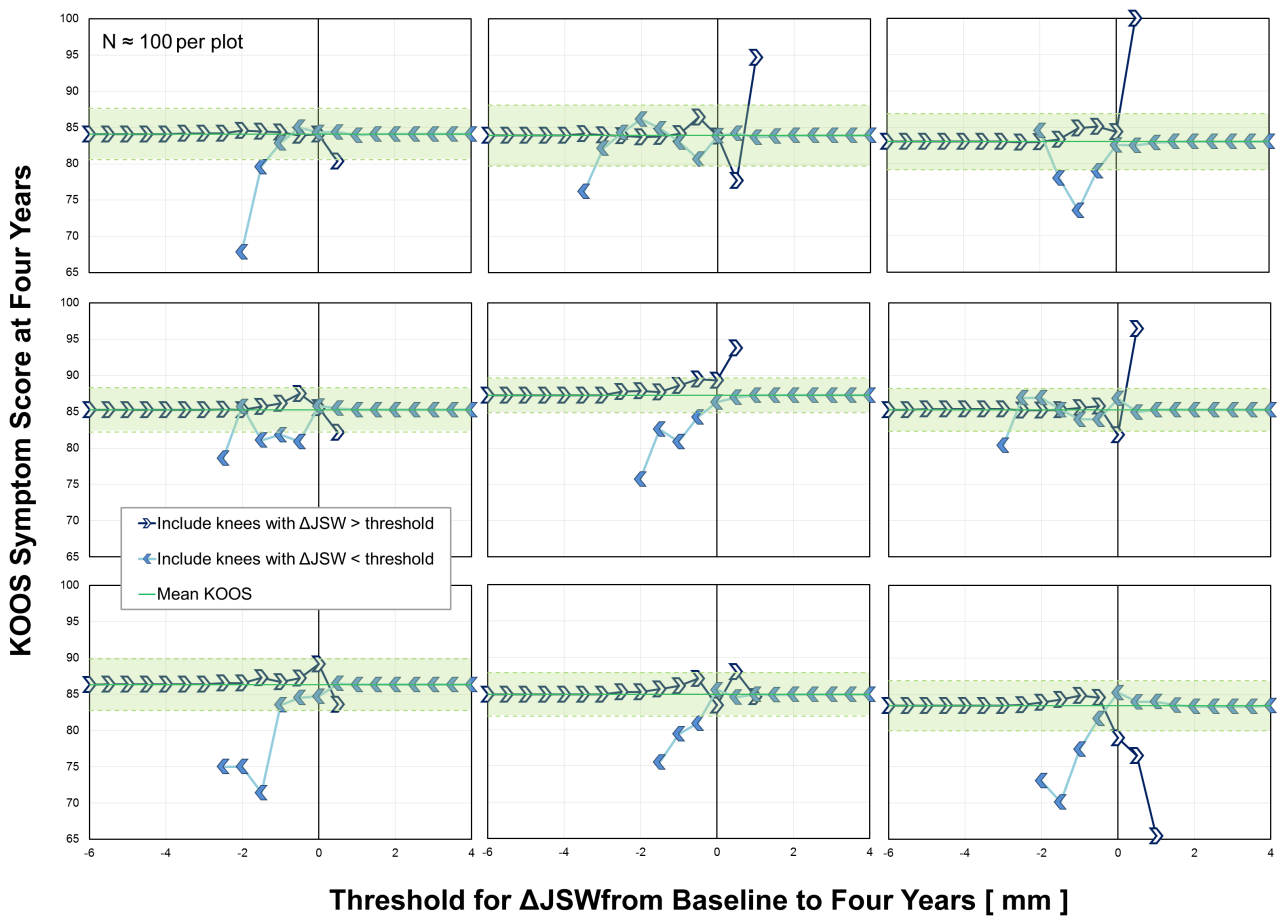
Application to Small Sample Sizes Graphical analysis of nine randomly selected subsamples of the data representing approximately 2.5% of the available data (N = 86 to 109 per plot). Threshold limit graphs aid in visualization of data trends even for small sample sizes and provide an additional option for understanding and interpreting data. Abbreviations: KOOS, Knee Osteoarthritis Outcome Score; ΔJSW, change in joint space width.

The threshold limit graph can also be helpful for the analysis of small datasets. To illustrate this, data for the 4,202 knees were randomly divided into 42 subsamples using a random number generator, and then each of those subsamples (~2.5% of the data) was plotted using the threshold limit technique. Figure 4 provides data for the first nine subsamples, which show similar trends to those observed in the entire population. Note that the 95% confidence interval for the mean for the subsamples (Figure 4) is wider than that of the entire population (Figure 2), which is a consequence of the smaller sample size. As with all graphical and statistical approaches to visualizing and analyzing data, a small sample size may or may not provide similar results to those that would be found using a large sample size. Nevertheless, a threshold limit graph of data from a small study could potentially help in the design of a larger study and offers an alternative and additional method to identify trends in the data.

## Discussion

Multiple approaches are available to understand how specific variables may affect an outcome [6]. A graphical approach such as the threshold limit graph may help to isolate the effect of individual variables by systematically including/excluding samples based on threshold values and comparing outcomes to the population mean. This information may be helpful in designing subsequent studies and in understanding the relative importance of a variable in patient management. Threshold limit graphs allow the user to estimate the effects on subsequent study outcomes when setting thresholds for variables for patient inclusion and/or exclusion criteria.

There has long been an interest in identifying any possible association between change in the knee joint space and knee outcomes [7-8]. A previous study showed that subjectively graded joint space narrowing was a risk factor for frequent pain in subjects that had frequent knee pain in one knee but not in the contralateral knee [9]. However, other investigators have not been able to document an association between joint space narrowing and symptoms [10-11]. A re-analysis of the data using the threshold limit graphical approach described in this paper may provide additional insights into the association between change in the knee joint space and outcomes in the OAI study, other knee studies, and eventually in clinical practice.

Although this graphical approach to understanding data requires an independent variable that is reported on a continuous scale, the dependent variable may be continuous or binary. When using a continuous variable, the average value can be plotted on the y-axis for specific subsets of the data. When using a binary dependent variable, the proportion of subjects with a positive outcome can be plotted on the Y-axis for each of a range of subgroups defined by threshold levels of the independent variable.

As with most statistical testing, a large sample size is desirable when using threshold-limit graphs. Application of the threshold-limit graph to a small sample of data may fail to show relationship between independent and dependent variables that would be seen with a larger sample size.

## Conclusions

There are both orthopedic and non-orthopedic problems where multiple factors may affect an outcome. By focusing on how a dependent variable is affected when data are included or excluded based on a threshold level of the independent variable, the threshold-limit approach to graphically displaying data may help to better understand associations between independent variables and outcomes.

Based on our analysis of the OAI data, there is a significant correlation between ΔJSW from baseline to four years and KOOS symptom subscores at four years for patients with specific ΔJSW characteristics. Patients with a loss of joint space of 0.5 mm or more had significantly lower KOOS symptom subscores than the average population. In contrast, patients with an increase in joint space of 1.5 mm or more had significantly higher KOOS symptom subscores than the average population.
